# Triclinic modification of *N*-[(1,1-di­methyl­ethoxy)carbon­yl]-3-[(*R*)-prop-2-en-1-ylsulfin­yl]-(*R*)-alanine ethyl ester at 120 (1) K

**DOI:** 10.1107/S1600536809019011

**Published:** 2009-05-23

**Authors:** Suneel P. Singh, Marcus J. Verdu, Alan J. Lough, Adrian L. Schwan

**Affiliations:** a Department of Chemistry, University of Guelph, Guelph, Ontario, Canada N1G 2W1; b Department of Chemistry, University of Toronto, Toronto, Ontario, Canada M5S 3H6

## Abstract

There are two independent mol­ecules in the asymmetric unit of the title compound, C_13_H_23_NO_5_S. In the crystal structure, inter­molecular N—H⋯O hydrogen bonds link mol­ecules into two independent one-dimensional chains along [100]. The crystal studied was found to be a non-merohedral twin with a ratio of 0.615 (6):0.385 (1) for the refined components. At 200 (1) K [Singh *et al.* (2009 ▶). *Acta Cryst*. E**65**, o1385–o1386] the crystal structure of the title compound contains one disordered mol­ecule in the asymmetric unit of a monoclinic unit cell.

## Related literature

For the crystal structure of the monlinic modification of the title compound at 200 (1) K and background information, see the preceding paper: Singh *et al.* (2009 ▶).
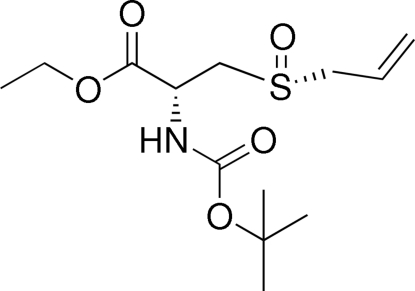


## Experimental

### 

#### Crystal data

C_13_H_23_NO_5_S
*M* *_r_* = 305.38
Triclinic, 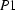

*a* = 5.1483 (5) Å
*b* = 11.6600 (15) Å
*c* = 13.6510 (19) Å
α = 88.884 (6)°
β = 82.681 (8)°
γ = 87.306 (8)°
*V* = 811.81 (17) Å^3^
*Z* = 2
Mo *K*α radiation
μ = 0.22 mm^−1^
*T* = 120 K
0.38 × 0.12 × 0.12 mm

#### Data collection

Nonius KappaCCD diffractometer
Absorption correction: multi-scan (*SORTAV*; Blessing, 1995 ▶) *T*_min_ = 0.561, *T*_max_ = 0.975
4279 measured reflections
4279 independent reflections
3526 reflections with *I* > 2σ(*I*)
*R*_int_ = 0.12

#### Refinement

*R*[*F* ^2^ > 2σ(*F* ^2^)] = 0.093
*wR*(*F* ^2^) = 0.261
*S* = 1.08
4279 reflections
362 parameters
3 restraints
H-atom parameters constrained
Δρ_max_ = 0.76 e Å^−3^
Δρ_min_ = −0.50 e Å^−3^
Absolute structure: Flack (1983 ▶), 1709 Friedel pairs
Flack parameter: −0.12 (18)

### 

Data collection: *COLLECT* (Nonius, 2002 ▶); cell refinement: *DENZO-SMN* (Otwinowski & Minor, 1997 ▶); data reduction: *DENZO-SMN*; program(s) used to solve structure: *SIR92* (Altomare *et al.*, 1994 ▶); program(s) used to refine structure: *SHELXTL* (Sheldrick, 2008 ▶); molecular graphics: *PLATON* (Spek, 2009 ▶); software used to prepare material for publication: *SHELXTL*.

## Supplementary Material

Crystal structure: contains datablocks global, I. DOI: 10.1107/S1600536809019011/hb2979sup1.cif

Structure factors: contains datablocks I. DOI: 10.1107/S1600536809019011/hb2979Isup2.hkl

Additional supplementary materials:  crystallographic information; 3D view; checkCIF report

## Figures and Tables

**Table 1:** Hydrogen-bond geometry (Å, °)

*D*—H⋯*A*	*D*—H	H⋯*A*	*D*⋯*A*	*D*—H⋯*A*
N1*A*—H1*AC*⋯O4*A*^i^	0.88	2.19	2.913 (9)	139
N1*B*—H1*BC*⋯O4*B*^ii^	0.88	2.20	2.904 (9)	137

Symmetry codes: (i) 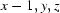; (ii) 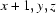.

**Fig. 1.**
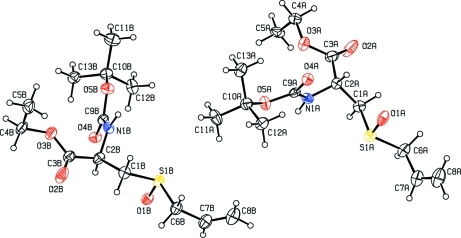
The asymmetric of (I): displacement elllipsoids are drawn at the 30% probabilty level.

**Fig. 2.**
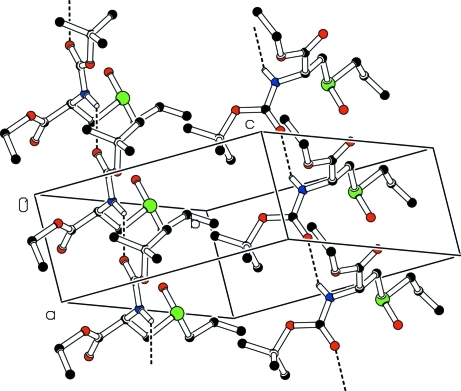
Part of the crystal structure of (I) showing hydrogen bonds as dashed lines.

**Fig. 3.**
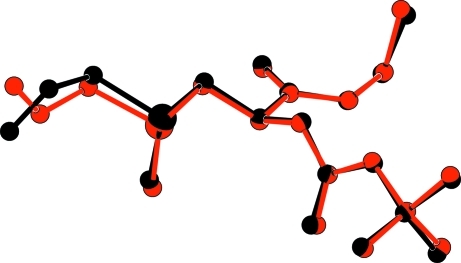
Overlay of both independent molecules of the title compound. Molecule A is drawn in black.

## supplementary crystallographic information

### Comment

For background information on the title compound see the previous paper (Singh *et al.*, 2009).

The asymmetric unit of the title compound is shown in Fig. 1. It contains two indpendent moleclues [A and B] which have essentially the same conformation apart from the orientation of the propenyl groups (see Fig. 3). This difference is reflected in the the values of the S1—C6—C7—C8 torsion angles for molecules A and B which are -89.5 (13) and 112.2 (13)°, respectively. Data for the title compound were also collected at 200 (1) K and the crystal structure solves and refines in the monoclinic space group *P*2_1_ (Singh *et al.*, 2009) with one molecule in the asymmetric unit and a disordered propenyl group. The torsion angles for the S1—C6—C7—C8 sequence of atoms in the major and minor components of the disorder are -99 (1) and 107 (3). The same crystal was used for both determinations. In the crystal structure, intermolecular hydrogen bonds link molecules into two independent one-dimensional chains along [100] (Table 1, Fig. 2).

### Experimental

For the synthetic procedure, see: Singh *et al.* (2009).

### Refinement

Hydrogen atoms were placed in calculated positions with C—H = 0.95–0.99; N—H = 0.88 Å and refined as riding with *U*_iso_(H) = 1.2*U*_eq_(C, N) or 1.5*U*_eq_(methyl C). The crystal is a non-merohedral twin: an analysis using *PLATON* (Spek, 2009) gave the twin law -1 0 0, -0.205 1 - 0.023, 0 0 - 1 with the ratio of twin components being 0.615 (6):0.385 (1).

### Crystal data

**Table:**

C_13_H_23_NO_5_S	*Z* = 2
*M**_r_* = 305.38	*F*(000) = 328
Triclinic, *P*1	*D*_x_ = 1.249 Mg m^−^^3^
Hall symbol: P 1	Mo *K*α radiation, λ = 0.71073 Å
*a* = 5.1483 (5) Å	Cell parameters from 4279 reflections
*b* = 11.6600 (15) Å	θ = 3.0–25.0°
*c* = 13.6510 (19) Å	µ = 0.22 mm^−^^1^
α = 88.884 (6)°	*T* = 120 K
β = 82.681 (8)°	Needle, colourless
γ = 87.306 (8)°	0.38 × 0.12 × 0.12 mm
*V* = 811.81 (17) Å^3^	

### Data collection

**Table:**

Nonius KappaCCD diffractometer	4279 independent reflections
Radiation source: fine-focus sealed tube	3526 reflections with *I* > 2σ(*I*)
graphite	*R*_int_ = 0.12
Detector resolution: 9 pixels mm^-1^	θ_max_ = 25.0°, θ_min_ = 3.0°
φ scans and ω scans with κ offsets	*h* = −6→6
Absorption correction: multi-scan (*SORTAV*; Blessing, 1995)	*k* = −13→13
*T*_min_ = 0.561, *T*_max_ = 0.975	*l* = −15→16
4279 measured reflections	

### Refinement

**Table:**

Refinement on *F*^2^	Secondary atom site location: difference Fourier map
Least-squares matrix: full	Hydrogen site location: inferred from neighbouring sites
*R*[*F*^2^ > 2σ(*F*^2^)] = 0.093	H-atom parameters constrained
*wR*(*F*^2^) = 0.261	*w* = 1/[σ^2^(*F*_o_^2^) + (0.1506*P*)^2^ + 0.8783*P*] where *P* = (*F*_o_^2^ + 2*F*_c_^2^)/3
*S* = 1.08	(Δ/σ)_max_ = 0.003
4279 reflections	Δρ_max_ = 0.76 e Å^−^^3^
362 parameters	Δρ_min_ = −0.49 e Å^−^^3^
3 restraints	Absolute structure: Flack (1983), 1709 Friedel pairs
Primary atom site location: structure-invariant direct methods	Flack parameter: −0.12 (18)

### Special details

**Table:**

Experimental. Absorption correction: multi-scan from symmetry-related measurements (SORTAV; Blessing, 1995)
Geometry. All e.s.d.'s (except the e.s.d. in the dihedral angle between two l.s. planes) are estimated using the full covariance matrix. The cell e.s.d.'s are taken into account individually in the estimation of e.s.d.'s in distances, angles and torsion angles; correlations between e.s.d.'s in cell parameters are only used when they are defined by crystal symmetry. An approximate (isotropic) treatment of cell e.s.d.'s is used for estimating e.s.d.'s involving l.s. planes.
Refinement. Refinement of *F*^2^ against ALL reflections. The weighted *R*-factor *wR* and goodness of fit *S* are based on *F*^2^, conventional *R*-factors *R* are based on *F*, with *F* set to zero for negative *F*^2^. The threshold expression of *F*^2^ > σ(*F*^2^) is used only for calculating *R*-factors(gt) *etc*. and is not relevant to the choice of reflections for refinement. *R*-factors based on *F*^2^ are statistically about twice as large as those based on *F*, and *R*- factors based on ALL data will be even larger.

### Fractional atomic coordinates and isotropic or equivalent isotropic displacement parameters (Å^2^)

**Table:**

	*x*	*y*	*z*	*U*_iso_*/*U*_eq_	
S1A	0.2491 (4)	0.89719 (19)	0.70483 (18)	0.0441 (7)	
O1A	0.5083 (12)	0.9001 (5)	0.7397 (6)	0.050 (2)	
O2A	−0.046 (3)	0.6226 (7)	0.9264 (8)	0.115 (4)	
O3A	0.1256 (13)	0.4856 (5)	0.8249 (6)	0.0507 (17)	
O4A	0.7047 (11)	0.5997 (5)	0.6652 (5)	0.0413 (15)	
O5A	0.4606 (12)	0.5281 (6)	0.5530 (5)	0.0463 (16)	
N1A	0.2589 (14)	0.6302 (6)	0.6753 (6)	0.0383 (18)	
H1AC	0.1245	0.6303	0.6415	0.046*	
C1A	0.0703 (18)	0.7904 (8)	0.7809 (8)	0.042 (2)	
H1AA	0.0412	0.8158	0.8504	0.051*	
H1AB	−0.1031	0.7821	0.7583	0.051*	
C2A	0.2212 (19)	0.6762 (8)	0.7748 (8)	0.042 (2)	
H2AA	0.3990	0.6892	0.7934	0.051*	
C3A	0.088 (2)	0.5935 (8)	0.8518 (9)	0.051 (3)	
C4A	−0.010 (2)	0.3966 (9)	0.8887 (8)	0.045 (2)	
H4AA	0.1043	0.3259	0.8886	0.054*	
H4AB	−0.0465	0.4243	0.9574	0.054*	
C5A	−0.256 (2)	0.3713 (10)	0.8523 (8)	0.052 (3)	
H5AA	−0.3435	0.3120	0.8943	0.078*	
H5AB	−0.2186	0.3438	0.7843	0.078*	
H5AC	−0.3694	0.4412	0.8537	0.078*	
C6A	0.058 (2)	1.0212 (9)	0.7578 (10)	0.053 (3)	
H6AA	−0.1303	1.0101	0.7560	0.063*	
H6AB	0.0870	1.0286	0.8277	0.063*	
C7A	0.135 (2)	1.1285 (9)	0.7021 (10)	0.060 (3)	
H7A	0.0429	1.1508	0.6483	0.072*	
C8A	0.317 (3)	1.1927 (11)	0.7218 (13)	0.081 (4)	
H8A1	0.4137	1.1730	0.7750	0.097*	
H8A2	0.3554	1.2598	0.6831	0.097*	
C9A	0.4911 (15)	0.5880 (7)	0.6343 (7)	0.0332 (19)	
C10A	0.6878 (18)	0.4846 (8)	0.4849 (7)	0.045 (2)	
C11A	0.558 (2)	0.4260 (10)	0.4071 (9)	0.057 (3)	
H11A	0.4571	0.4832	0.3723	0.086*	
H11B	0.4412	0.3685	0.4387	0.086*	
H11C	0.6933	0.3882	0.3598	0.086*	
C12A	0.849 (2)	0.5805 (8)	0.4435 (8)	0.051 (3)	
H12A	0.7395	0.6356	0.4098	0.077*	
H12B	0.9941	0.5504	0.3964	0.077*	
H12C	0.9183	0.6190	0.4971	0.077*	
C13A	0.846 (2)	0.3923 (9)	0.5393 (9)	0.052 (3)	
H13A	0.9297	0.4291	0.5904	0.077*	
H13B	0.9812	0.3550	0.4918	0.077*	
H13C	0.7277	0.3348	0.5700	0.077*	
S1B	0.1520 (4)	0.40207 (18)	0.18589 (17)	0.0407 (6)	
O1B	−0.1088 (12)	0.4057 (5)	0.1524 (5)	0.0425 (17)	
O2B	0.467 (2)	0.1249 (6)	−0.0308 (7)	0.084 (3)	
O3B	0.3134 (13)	−0.0110 (5)	0.0748 (5)	0.0487 (17)	
O4B	−0.2688 (11)	0.1054 (5)	0.2279 (5)	0.0411 (15)	
O5B	−0.0182 (11)	0.0315 (6)	0.3428 (5)	0.0444 (16)	
N1B	0.1704 (14)	0.1343 (6)	0.2209 (6)	0.0372 (18)	
H1BC	0.3029	0.1340	0.2557	0.045*	
C1B	0.3469 (17)	0.2925 (8)	0.1143 (8)	0.041 (2)	
H1BA	0.3831	0.3186	0.0447	0.050*	
H1BB	0.5167	0.2789	0.1404	0.050*	
C2B	0.2017 (18)	0.1810 (8)	0.1194 (8)	0.041 (2)	
H2BA	0.0231	0.1986	0.1000	0.050*	
C3B	0.342 (2)	0.0972 (8)	0.0474 (8)	0.047 (2)	
C4B	0.455 (2)	−0.1020 (9)	0.0122 (10)	0.054 (3)	
H4BA	0.4769	−0.0765	−0.0579	0.065*	
H4BB	0.3548	−0.1727	0.0179	0.065*	
C5B	0.718 (2)	−0.1254 (10)	0.0464 (9)	0.059 (3)	
H5BA	0.8162	−0.1850	0.0055	0.089*	
H5BB	0.6947	−0.1515	0.1155	0.089*	
H5BC	0.8158	−0.0549	0.0407	0.089*	
C6B	0.322 (2)	0.5239 (8)	0.1283 (9)	0.045 (2)	
H6BA	0.5068	0.5193	0.1414	0.054*	
H6BB	0.3198	0.5224	0.0559	0.054*	
C7B	0.190 (2)	0.6333 (9)	0.1687 (10)	0.056 (3)	
H7B	0.0191	0.6528	0.1529	0.067*	
C8B	0.293 (3)	0.7024 (11)	0.2231 (11)	0.071 (4)	
H8B1	0.4639	0.6856	0.2403	0.086*	
H8B2	0.1980	0.7705	0.2462	0.086*	
C9B	−0.0584 (16)	0.0921 (7)	0.2610 (7)	0.035 (2)	
C10B	−0.2460 (17)	−0.0139 (8)	0.4090 (8)	0.044 (2)	
C11B	−0.107 (2)	−0.0749 (11)	0.4897 (9)	0.061 (3)	
H11D	−0.0214	−0.0182	0.5253	0.092*	
H11E	0.0257	−0.1310	0.4593	0.092*	
H11F	−0.2352	−0.1145	0.5359	0.092*	
C12B	−0.4219 (18)	0.0843 (9)	0.4501 (8)	0.052 (3)	
H12D	−0.5083	0.1214	0.3971	0.078*	
H12E	−0.3177	0.1402	0.4787	0.078*	
H12F	−0.5547	0.0557	0.5014	0.078*	
C13B	−0.3820 (19)	−0.1009 (8)	0.3539 (8)	0.047 (3)	
H13D	−0.4722	−0.0611	0.3031	0.070*	
H13E	−0.5098	−0.1404	0.4004	0.070*	
H13F	−0.2514	−0.1571	0.3226	0.070*	

### Atomic displacement parameters (Å^2^)

**Table:**

	*U*^11^	*U*^22^	*U*^33^	*U*^12^	*U*^13^	*U*^23^
S1A	0.0443 (15)	0.0370 (13)	0.0497 (18)	0.0002 (10)	−0.0016 (12)	−0.0016 (11)
O1A	0.034 (4)	0.035 (4)	0.078 (6)	0.003 (3)	0.000 (4)	−0.011 (3)
O2A	0.223 (12)	0.033 (4)	0.065 (7)	0.014 (5)	0.062 (8)	0.006 (4)
O3A	0.055 (4)	0.038 (4)	0.058 (5)	−0.005 (3)	−0.003 (3)	−0.007 (3)
O4A	0.035 (3)	0.040 (3)	0.049 (4)	−0.001 (3)	−0.006 (3)	−0.003 (3)
O5A	0.041 (4)	0.055 (4)	0.043 (4)	−0.004 (3)	−0.003 (3)	−0.015 (3)
N1A	0.034 (4)	0.046 (4)	0.036 (5)	0.002 (3)	−0.011 (3)	0.001 (4)
C1A	0.036 (5)	0.045 (5)	0.045 (6)	0.000 (4)	−0.003 (4)	0.002 (5)
C2A	0.050 (6)	0.034 (5)	0.043 (6)	0.001 (4)	−0.008 (4)	−0.003 (4)
C3A	0.058 (6)	0.043 (6)	0.049 (7)	0.003 (4)	−0.003 (5)	0.008 (5)
C4A	0.046 (6)	0.050 (6)	0.040 (6)	−0.002 (4)	−0.004 (5)	0.005 (5)
C5A	0.060 (7)	0.059 (6)	0.039 (6)	−0.019 (5)	−0.010 (5)	0.019 (5)
C6A	0.055 (6)	0.038 (5)	0.064 (8)	−0.004 (4)	−0.003 (5)	−0.001 (5)
C7A	0.057 (7)	0.051 (7)	0.071 (9)	−0.007 (5)	0.001 (6)	−0.012 (6)
C8A	0.073 (8)	0.061 (8)	0.105 (12)	−0.016 (6)	0.016 (8)	−0.019 (8)
C9A	0.029 (5)	0.028 (4)	0.043 (6)	−0.001 (3)	−0.009 (4)	0.006 (4)
C10A	0.043 (5)	0.045 (5)	0.044 (6)	0.002 (4)	−0.001 (4)	−0.001 (4)
C11A	0.044 (6)	0.083 (8)	0.044 (7)	0.004 (5)	−0.004 (5)	−0.021 (6)
C12A	0.054 (6)	0.043 (5)	0.056 (7)	0.001 (4)	−0.001 (5)	0.003 (5)
C13A	0.046 (6)	0.045 (6)	0.061 (8)	−0.001 (4)	0.006 (5)	−0.004 (5)
S1B	0.0410 (14)	0.0346 (13)	0.0462 (17)	−0.0050 (9)	−0.0031 (11)	0.0014 (10)
O1B	0.039 (4)	0.035 (4)	0.053 (5)	−0.005 (3)	−0.004 (3)	0.002 (3)
O2B	0.151 (9)	0.035 (4)	0.057 (6)	−0.020 (4)	0.034 (6)	0.001 (4)
O3B	0.056 (4)	0.035 (4)	0.053 (5)	0.002 (3)	−0.001 (3)	−0.002 (3)
O4B	0.033 (3)	0.043 (4)	0.047 (4)	−0.003 (3)	−0.006 (3)	0.008 (3)
O5B	0.038 (4)	0.051 (4)	0.045 (4)	−0.008 (3)	−0.006 (3)	0.009 (3)
N1B	0.032 (4)	0.045 (4)	0.036 (5)	−0.009 (3)	−0.008 (3)	−0.002 (4)
C1B	0.031 (5)	0.042 (5)	0.051 (7)	−0.002 (4)	−0.003 (4)	−0.004 (5)
C2B	0.035 (5)	0.035 (5)	0.055 (7)	−0.005 (4)	−0.007 (4)	0.003 (4)
C3B	0.054 (6)	0.035 (5)	0.052 (7)	−0.006 (4)	−0.011 (5)	0.004 (5)
C4B	0.074 (7)	0.027 (5)	0.059 (8)	−0.003 (4)	0.005 (6)	0.000 (5)
C5B	0.060 (7)	0.054 (6)	0.061 (8)	0.010 (5)	−0.002 (5)	−0.015 (6)
C6B	0.040 (5)	0.040 (5)	0.054 (7)	−0.006 (4)	−0.002 (4)	−0.002 (4)
C7B	0.049 (6)	0.045 (6)	0.073 (9)	−0.006 (5)	−0.003 (6)	−0.001 (6)
C8B	0.066 (7)	0.050 (7)	0.094 (11)	−0.009 (5)	0.008 (7)	−0.009 (7)
C9B	0.034 (5)	0.031 (4)	0.039 (6)	−0.005 (3)	−0.001 (4)	0.003 (4)
C10B	0.042 (5)	0.044 (5)	0.044 (6)	−0.005 (4)	−0.002 (4)	0.005 (4)
C11B	0.054 (6)	0.077 (8)	0.050 (7)	−0.013 (5)	0.002 (5)	0.017 (6)
C12B	0.042 (5)	0.063 (7)	0.049 (7)	−0.011 (5)	0.003 (5)	−0.013 (5)
C13B	0.048 (6)	0.043 (6)	0.048 (7)	−0.006 (4)	0.000 (5)	0.005 (5)

### Geometric parameters (Å, °)

**Table:**

S1A—O1A	1.475 (7)	S1B—O1B	1.471 (7)
S1A—C1A	1.814 (10)	S1B—C1B	1.809 (10)
S1A—C6A	1.818 (11)	S1B—C6B	1.816 (11)
O2A—C3A	1.200 (14)	O2B—C3B	1.220 (13)
O3A—C3A	1.316 (12)	O3B—C3B	1.319 (12)
O3A—C4A	1.483 (13)	O3B—C4B	1.481 (13)
O4A—C9A	1.241 (10)	O4B—C9B	1.228 (10)
O5A—C9A	1.353 (11)	O5B—C9B	1.344 (11)
O5A—C10A	1.475 (11)	O5B—C10B	1.497 (11)
N1A—C9A	1.330 (11)	N1B—C9B	1.345 (11)
N1A—C2A	1.456 (13)	N1B—C2B	1.471 (13)
N1A—H1AC	0.8800	N1B—H1BC	0.8800
C1A—C2A	1.508 (12)	C1B—C2B	1.526 (13)
C1A—H1AA	0.9900	C1B—H1BA	0.9900
C1A—H1AB	0.9900	C1B—H1BB	0.9900
C2A—C3A	1.532 (15)	C2B—C3B	1.495 (14)
C2A—H2AA	1.0000	C2B—H2BA	1.0000
C4A—C5A	1.461 (14)	C4B—C5B	1.500 (16)
C4A—H4AA	0.9900	C4B—H4BA	0.9900
C4A—H4AB	0.9900	C4B—H4BB	0.9900
C5A—H5AA	0.9800	C5B—H5BA	0.9800
C5A—H5AB	0.9800	C5B—H5BB	0.9800
C5A—H5AC	0.9800	C5B—H5BC	0.9800
C6A—C7A	1.494 (17)	C6B—C7B	1.497 (14)
C6A—H6AA	0.9900	C6B—H6BA	0.9900
C6A—H6AB	0.9900	C6B—H6BB	0.9900
C7A—C8A	1.283 (17)	C7B—C8B	1.282 (17)
C7A—H7A	0.9500	C7B—H7B	0.9500
C8A—H8A1	0.9500	C8B—H8B1	0.9500
C8A—H8A2	0.9500	C8B—H8B2	0.9500
C10A—C12A	1.483 (14)	C10B—C12B	1.498 (13)
C10A—C11A	1.513 (14)	C10B—C13B	1.521 (14)
C10A—C13A	1.554 (14)	C10B—C11B	1.535 (15)
C11A—H11A	0.9800	C11B—H11D	0.9800
C11A—H11B	0.9800	C11B—H11E	0.9800
C11A—H11C	0.9800	C11B—H11F	0.9800
C12A—H12A	0.9800	C12B—H12D	0.9800
C12A—H12B	0.9800	C12B—H12E	0.9800
C12A—H12C	0.9800	C12B—H12F	0.9800
C13A—H13A	0.9800	C13B—H13D	0.9800
C13A—H13B	0.9800	C13B—H13E	0.9800
C13A—H13C	0.9800	C13B—H13F	0.9800
			
O1A—S1A—C1A	106.2 (4)	O1B—S1B—C1B	106.4 (4)
O1A—S1A—C6A	106.3 (5)	O1B—S1B—C6B	106.8 (4)
C1A—S1A—C6A	96.2 (5)	C1B—S1B—C6B	96.5 (5)
C3A—O3A—C4A	118.2 (8)	C3B—O3B—C4B	118.7 (8)
C9A—O5A—C10A	121.6 (7)	C9B—O5B—C10B	120.0 (6)
C9A—N1A—C2A	121.8 (8)	C9B—N1B—C2B	120.8 (8)
C9A—N1A—H1AC	119.1	C9B—N1B—H1BC	119.6
C2A—N1A—H1AC	119.1	C2B—N1B—H1BC	119.6
C2A—C1A—S1A	110.4 (7)	C2B—C1B—S1B	110.2 (6)
C2A—C1A—H1AA	109.6	C2B—C1B—H1BA	109.6
S1A—C1A—H1AA	109.6	S1B—C1B—H1BA	109.6
C2A—C1A—H1AB	109.6	C2B—C1B—H1BB	109.6
S1A—C1A—H1AB	109.6	S1B—C1B—H1BB	109.6
H1AA—C1A—H1AB	108.1	H1BA—C1B—H1BB	108.1
N1A—C2A—C1A	112.7 (8)	N1B—C2B—C3B	112.1 (8)
N1A—C2A—C3A	113.1 (8)	N1B—C2B—C1B	110.4 (8)
C1A—C2A—C3A	109.1 (8)	C3B—C2B—C1B	109.9 (8)
N1A—C2A—H2AA	107.2	N1B—C2B—H2BA	108.1
C1A—C2A—H2AA	107.2	C3B—C2B—H2BA	108.1
C3A—C2A—H2AA	107.2	C1B—C2B—H2BA	108.1
O2A—C3A—O3A	123.0 (10)	O2B—C3B—O3B	122.5 (10)
O2A—C3A—C2A	124.6 (9)	O2B—C3B—C2B	123.8 (9)
O3A—C3A—C2A	112.3 (9)	O3B—C3B—C2B	113.6 (9)
C5A—C4A—O3A	109.9 (8)	O3B—C4B—C5B	108.1 (9)
C5A—C4A—H4AA	109.7	O3B—C4B—H4BA	110.1
O3A—C4A—H4AA	109.7	C5B—C4B—H4BA	110.1
C5A—C4A—H4AB	109.7	O3B—C4B—H4BB	110.1
O3A—C4A—H4AB	109.7	C5B—C4B—H4BB	110.1
H4AA—C4A—H4AB	108.2	H4BA—C4B—H4BB	108.4
C4A—C5A—H5AA	109.5	C4B—C5B—H5BA	109.5
C4A—C5A—H5AB	109.5	C4B—C5B—H5BB	109.5
H5AA—C5A—H5AB	109.5	H5BA—C5B—H5BB	109.5
C4A—C5A—H5AC	109.5	C4B—C5B—H5BC	109.5
H5AA—C5A—H5AC	109.5	H5BA—C5B—H5BC	109.5
H5AB—C5A—H5AC	109.5	H5BB—C5B—H5BC	109.5
C7A—C6A—S1A	110.9 (8)	C7B—C6B—S1B	109.7 (8)
C7A—C6A—H6AA	109.5	C7B—C6B—H6BA	109.7
S1A—C6A—H6AA	109.5	S1B—C6B—H6BA	109.7
C7A—C6A—H6AB	109.5	C7B—C6B—H6BB	109.7
S1A—C6A—H6AB	109.5	S1B—C6B—H6BB	109.7
H6AA—C6A—H6AB	108.1	H6BA—C6B—H6BB	108.2
C8A—C7A—C6A	124.6 (14)	C8B—C7B—C6B	124.5 (11)
C8A—C7A—H7A	117.7	C8B—C7B—H7B	117.8
C6A—C7A—H7A	117.7	C6B—C7B—H7B	117.8
C7A—C8A—H8A1	120.0	C7B—C8B—H8B1	120.0
C7A—C8A—H8A2	120.0	C7B—C8B—H8B2	120.0
H8A1—C8A—H8A2	120.0	H8B1—C8B—H8B2	120.0
O4A—C9A—N1A	126.2 (8)	O4B—C9B—O5B	125.5 (7)
O4A—C9A—O5A	124.3 (7)	O4B—C9B—N1B	125.9 (8)
N1A—C9A—O5A	109.5 (7)	O5B—C9B—N1B	108.7 (7)
O5A—C10A—C12A	110.6 (7)	O5B—C10B—C12B	109.5 (7)
O5A—C10A—C11A	102.4 (7)	O5B—C10B—C13B	109.9 (8)
C12A—C10A—C11A	112.5 (9)	C12B—C10B—C13B	114.2 (8)
O5A—C10A—C13A	109.3 (8)	O5B—C10B—C11B	101.2 (7)
C12A—C10A—C13A	113.0 (8)	C12B—C10B—C11B	111.3 (9)
C11A—C10A—C13A	108.6 (9)	C13B—C10B—C11B	110.0 (9)
C10A—C11A—H11A	109.5	C10B—C11B—H11D	109.5
C10A—C11A—H11B	109.5	C10B—C11B—H11E	109.5
H11A—C11A—H11B	109.5	H11D—C11B—H11E	109.5
C10A—C11A—H11C	109.5	C10B—C11B—H11F	109.5
H11A—C11A—H11C	109.5	H11D—C11B—H11F	109.5
H11B—C11A—H11C	109.5	H11E—C11B—H11F	109.5
C10A—C12A—H12A	109.5	C10B—C12B—H12D	109.5
C10A—C12A—H12B	109.5	C10B—C12B—H12E	109.5
H12A—C12A—H12B	109.5	H12D—C12B—H12E	109.5
C10A—C12A—H12C	109.5	C10B—C12B—H12F	109.5
H12A—C12A—H12C	109.5	H12D—C12B—H12F	109.5
H12B—C12A—H12C	109.5	H12E—C12B—H12F	109.5
C10A—C13A—H13A	109.5	C10B—C13B—H13D	109.5
C10A—C13A—H13B	109.5	C10B—C13B—H13E	109.5
H13A—C13A—H13B	109.5	H13D—C13B—H13E	109.5
C10A—C13A—H13C	109.5	C10B—C13B—H13F	109.5
H13A—C13A—H13C	109.5	H13D—C13B—H13F	109.5
H13B—C13A—H13C	109.5	H13E—C13B—H13F	109.5

### Hydrogen-bond geometry (Å, °)

**Table:**

*D*—H···*A*	*D*—H	H···*A*	*D*···*A*	*D*—H···*A*
N1A—H1AC···O4A^i^	0.88	2.19	2.913 (9)	139
N1B—H1BC···O4B^ii^	0.88	2.20	2.904 (9)	137

Symmetry codes: (i) *x*−1, *y*, *z*; (ii) *x*+1, *y*, *z*.
